# Focal Adhesion Kinase and ROCK Signaling Are Switch-Like Regulators of Human Adipose Stem Cell Differentiation towards Osteogenic and Adipogenic Lineages

**DOI:** 10.1155/2018/2190657

**Published:** 2018-09-12

**Authors:** Laura Hyväri, Miina Ojansivu, Miia Juntunen, Kimmo Kartasalo, Susanna Miettinen, Sari Vanhatupa

**Affiliations:** ^1^Adult Stem Cell Research Group, BioMediTech Institute and Faculty of Medicine and Life Sciences, University of Tampere, Tampere, Finland; ^2^Science Center, Tampere University Hospital, Tampere, Finland; ^3^Computational Biology Group, BioMediTech Institute and Faculty of Medicine and Life Sciences, University of Tampere, Tampere, Finland; ^4^BioMediTech Institute and Faculty of Biomedical Sciences and Engineering, Tampere University of Technology, Tampere, Finland

## Abstract

Adipose tissue is an attractive stem cell source for soft and bone tissue engineering applications and stem cell therapies. The adipose-derived stromal/stem cells (ASCs) have a multilineage differentiation capacity that is regulated through extracellular signals. The cellular events related to cell adhesion and cytoskeleton have been suggested as central regulators of differentiation fate decision. However, the detailed knowledge of these molecular mechanisms in human ASCs remains limited. This study examined the significance of focal adhesion kinase (FAK), Rho-Rho-associated protein kinase (Rho-ROCK), and their downstream target extracellular signal-regulated kinase 1/2 (ERK1/2) on hASCs differentiation towards osteoblasts and adipocytes. Analyses of osteogenic markers *RUNX2A*, alkaline phosphatase, and matrix mineralization revealed an essential role of active FAK, ROCK, and ERK1/2 signaling for the osteogenesis of hASCs. Inhibition of these kinases with specific small molecule inhibitors diminished osteogenesis, while inhibition of FAK and ROCK activity led to elevation of adipogenic marker genes *AP2* and *LEP* and lipid accumulation implicating adipogenesis. This denotes to a switch-like function of FAK and ROCK signaling in the osteogenic and adipogenic fates of hASCs. On the contrary, inhibition of ERK1/2 kinase activity deceased adipogenic differentiation, indicating that activation of ERK signaling is required for both adipogenic and osteogenic potential. Our findings highlight the reciprocal role of cell adhesion mechanisms and actin dynamics in regulation of hASC lineage commitment. This study enhances the knowledge of molecular mechanisms dictating hASC differentiation and thus opens possibilities for more efficient control of hASC differentiation.

## 1. Introduction

Mesenchymal stem cells (MSCs) are multipotent adult stem cells that give rise to osteoblasts, adipocytes, and chondrocytes *in vitro*. MSCs can be harvested from multiple adult tissues, for example, bone marrow, adipose tissue, dental tissues, and umbilical cord [1]. MSCs derived from fat tissue, adipose-derived stromal/stem cells (ASCs), are increasingly used in regenerative medicine due to their desirable immunomodulatory properties and ease of harvest [1]. Regulation of MSC differentiation has been extensively studied, but the research has been mainly conducted with the bone marrow mesenchymal stem cells (BMSC) of human or rodent origin. Although the central transcription factors and signaling pathways are conserved between cell types and species, the extrapolation of these previous results to human ASCs cannot be made without reservation. It has been discovered that MSCs of different species are not fully comparable regarding their differentiation potential [2, 3] or immunosuppressive capacity [4]. Additionally, the differentiation potential of MSCs has been shown to vary even within species depending on the harvest site [5]. Thus, there is a need for *in vitro* studies elucidating the molecular mechanisms regulating differentiation potential specifically in human ASCs.

Self-renewal and differentiation of mesenchymal stem cells are tightly regulated by signals from the surrounding environment. Especially signals that regulate cell adhesion and cytoskeletal arrangements have been suggested to be important regulators of MSC differentiation [6]. Cells grow and function in association with extracellular matrix (ECM) components and respond to a wide range of external signals by converting their morphology, behavior, and fate decision accordingly [7–9]. One of the most important response mechanisms is based on the function of transmembrane adhesion receptors of the integrin family and integrin-based focal adhesion (FA) complexes. FAs work in the regulation of cytoskeletal networking and cellular signaling through a central mediator, focal adhesion kinase (FAK) [10]. FAK signaling functions through autophosphorylation of tyrosine 397 that induces interaction of FAK with Src, a nonreceptor tyrosine kinase that stabilizes as a response of this interaction and further phosphorylates other tyrosines of FAK. This leads to full activity of both kinases and subsequent activation of numerous intracellular pathways [11]. In mesenchymal stem cells (MSCs), FAK signaling is interconnected with various pathways including mitogen-activated kinases (MAPKs) and Rho-family GTPases RhoA, Rac, and Cdc42 [12].

The regulation of the cell cytoskeleton and morphology is primarily controlled by the RhoA-ROCK pathway [13], which sustains the integrity of the cytoskeleton by stimulating actomyosin contractility [14, 15]. ROCK isoforms are protein serine/threonine kinases that phosphorylate substrates such as myosin light chain (MLC) phosphatase to drive the assembly of the actin cytoskeleton [13]. The RhoA-ROCK signaling is also an important regulator of stem cell commitment [7, 16–18], and the cell shape determined by RhoA function has been proposed to be a major switch between adipogenic and osteogenic differentiation of human MSCs (hMSCs) [7]. In addition, ROCK signaling is related to the substrate stiffness-driven lineage commitment of MSCs through mechanosensing of the microenvironment via interplay with integrin-FAK signaling [19].

MAPK pathway component extracellular signal-regulated kinase 1/2 (ERK1/2) is linked to vital cellular functions such as proliferation, survival, apoptosis, motility, transcription, metabolism, and differentiation [20]. ERK1/2 has been shown to be a downstream effector of FAK-mediated signaling in MSCs [18, 21]. It has also been suggested as a mechanosensing protein, regulated by the RhoA-ROCK-mediated actin dynamics in hMSCs [22–24]. ERK1/2 activity is linked to the expression of osteogenic markers in hASCs [25]. However, the role of ERK signaling in the adipogenic differentiation fate differs depending on the experimental design and the cell type studied [25–28].

In previous studies, the cellular mechanisms of adhesion and cytoskeletal arrangements have been studied in multiple cell types and varying experimental conditions and configurations. In this study, our objective was to clarify the role of these mechanisms in the differentiation fate decision of adipose tissue-derived stem cells. The current study carefully analyzed the significance of FAK, ROCK, and ERK1/2 proteins in the adipogenic and osteogenic differentiation of hASCs. The key results demonstrated the reciprocal regulation of FAK and ROCK signaling in the interface of hASC osteogenesis and adipogenesis. Our results also consistently indicated that in hASCs, ERK1/2 activity is required for the full osteogenic and adipogenic potential. As a conclusion, our results suggested that ERK1/2 activation together with cell adhesion and actin regulation by FAK-RhoA-ROCK signaling are fine tuning regulators of hASC fate decision. This investigation enhanced the understanding of the signaling mechanisms governing stem cell commitment and gave insight for future development of *in vitro* models, tissue engineering constructs, and stem cell therapies.

## 2. Materials and Methods

### 2.1. Cell Isolation and Culture

The study was carried out in accordance with the Ethics Committee of the Pirkanmaa Hospital District, Tampere, Finland (ethical approval R15161). The hASCs were isolated from adipose tissue samples of six female donors (age, 44 ± 11 years, donor information in Table S1) with a written informed consent of the donors. Isolation of the stem cells was performed as described previously [29]. The isolated hASCs were maintained and expanded in human serum containing basic culture medium (BM) (composition in Table 1) and passaged after reaching 70–80% confluence.

### 2.2. Flow Cytometric Analysis of Surface Marker Expression

The cells were identified as MSCs by flow cytometry (FACSAria; BD Biosciences, Erembodegem, Belgium) at passage 1 to confirm the MSC immunophenotype of the cells. Cells were single stained using monoclonal antibodies against CD3-PE, CD14-PE-Cy7, CD19-PE-Cy7, CD45R0-APC, CD54-FITC, CD73-PE, CD90-APC (BD Biosciences, Franklin Lakes, NJ, USA), CD11a-APC, CD80-PE, CD86-PE, CD105-PE (R&D Systems, Minneapolis, MN, USA), CD34-APC, and HLA-DR-PE (ImmunoTools, Friesoythe, Germany). The FACS analysis was performed on 10,000 cells per sample and positive expression was defined as fluorescence level greater than 99% of the comparable unstained cell sample.

### 2.3. Osteogenic and Adipogenic Differentiation Cultures

Human ASCs were seeded into CellBIND polystyrene plates (Corning Inc., Corning, NY, USA) in BM prior to the experiments. Osteogenic and adipogenic inductions were initiated on the following day by introducing the osteogenic medium (OM) and adipogenic medium (AM) to the cells (compositions in Table 1). 0.25 mM IBMX (3-isobutyl-1-methylxanthine; Sigma-Aldrich, Saint Louis, MO, USA) was added to the adipogenic differentiation cultures upon first change of culture media. 5 nM Dexamethasone (DEX; Sigma-Aldrich) was applied to OM when used. Fresh differentiation media were applied to the cells twice a week during the experiments. As a control, the hASCs were cultured in BM condition. The experiments were conducted at passages 3–5.

### 2.4. Small Molecule Inhibitors

BM, OM, and AM were supplemented with small molecule inhibitors targeted to FAK, ROCK, and ERK1/2 proteins and added to the cell cultures. FAK and ROCK signaling were inhibited using PF-562271 (Selleck Chemicals, Houston, Texas, USA) and Y-27632 (Selleck Chemicals), respectively. Inhibition of ERK1/2 activation was conducted with PD98059 (Calbiochem/EMD Millipore, Billerica, Massachusetts, USA) which is a specific inhibitor of ERK1/2 upstream kinase mitogen-activated protein kinase 1 (MEK1). BM, OM, and AM conditions without the inhibitors were used as controls. Fresh media supplemented with the inhibitors were applied to the cells twice a week during the experiments.

### 2.5. Live/Dead Staining

The viability of the hASCs seeded 260 cells/cm^2^ in 24-well plate and cultured 7 days in BM, OM, or AM and left untreated (control) or treated with FAK inhibitor PF-562271, ROCK inhibitor Y-27632, or MEK/ERK inhibitor PD98059 was studied with LIVE/DEAD Viability/Cytotoxicity Kit (Molecular Probes; Thermo Fisher Scientific). The viable cells (green fluorescence) and dead cells (red fluorescence) were imaged using an Olympus microscope (IX51, Olympus) equipped with a fluorescence unit and camera (DP30BW, Olympus) with 4x magnification.

### 2.6. Fluorescence Staining of the Actin Cytoskeleton

The hASCs were cultured 7 days in BM, OM, or AM and left untreated (control) or treated with 2 *μ*M FAK inhibitor PF-562271, 15 *μ*M ROCK inhibitor Y-27632, and 30 *μ*M MEK/ERK inhibitor PD98059. The cells were fixed and permeabilized with 4% PFA (Sigma-Aldrich) supplemented with 0.1% Triton X-100 for 15 min at RT. Blocking was done with 1% bovine serum albumin (BSA; Sigma-Aldrich) for 1 h at +4°C. For actin staining, the cells were incubated in tetramethyl-rhodamine B isothiocyanate- (TRITC-) conjugated phalloidin (P1951; Sigma-Aldrich) for 45 min at RT followed by 4′,6-diamidino-2-phenylindole (DAPI, Sigma-Aldrich) staining to visualize the nuclei.

### 2.7. Cell Proliferation and Quantitative Analysis of Alkaline Phosphatase Activity

Cell proliferation of control and inhibitor-treated hASCs (seeded 260 cells/cm^2^ in 24-well plate) was assessed with CyQUANT cell proliferation assay (Molecular Probes; Thermo Fisher Scientific, Waltham, MA, USA) after 7 and 14 days of culture as described previously [29, 30]. The activity of alkaline phosphatase (ALP) was analyzed from the same cell lysates as cell proliferation as described previously [29].

### 2.8. Alizarin Red Staining and Quantification of Mineralization

The cells (seeded 260 cells/cm^2^ and cultured with control and inhibitor conditions) were stained with Alizarin Red (AR) after 14 and 21 days of culture for the analysis of mineralization. The staining was done as described previously [31]. Briefly, the cells were fixed with 70% ethanol, stained with 2% Alizarin Red S (pH 4.1–4.3; Sigma-Aldrich), and photographed after three washes with water and one with ethanol. Quantitative results were obtained by extracting the dye with 100 mM cetylpyridinium chloride (Sigma-Aldrich) for 3 hours and measuring the absorbances of the samples at 544 nm.

### 2.9. Oil Red O Staining

hASCs (seeded 260 cells/cm^2^ in 24-well plate) were cultured in BM, OM, and AM supplemented with inhibitor molecules for 21 days and stained with Oil Red O (ORO) staining, which indicates lipid droplet formation, as described previously [29]. Following ORO stain, the hASCs were counterstained with DAPI (Sigma-Aldrich; dilution 1 : 2000) for 5 minutes before the last washing steps. Fluorescence microscope images were taken with an Olympus microscope (IX51, Olympus, Tokyo, Japan) equipped with a fluorescence unit and a camera (DP30BW, Olympus).

### 2.10. Quantification of Lipid Formation

Lipid formation was quantified based on image analysis of samples stained with ORO and DAPI. Image quantification was performed with a custom analysis pipeline designed for CellProfiler (version 2.1.1, 64-bit Windows; http://www.cellprofiler.org [32]). Lipid maturation was assessed by applying a 10 *μ*m diameter threshold for lipid droplet clusters. See Supplemental Materials for a detailed description of the analysis pipeline.

### 2.11. qRT-PCR

The quantitative real-time reverse transcriptase polymerase chain reaction (qRT-PCR) analysis was performed after 7 and 14 days of culture (hASCs seeded 3160 cells/cm^2^ in 6-well plate) as described previously [33]. The expressions of *human runt-related transcription factor 2a (RUNX2A)*, *human adipocyte fatty acid-binding protein (FABP4 or AP2)*, and *human leptin* (*LEP)* were normalized with the expression of *human acidic ribosomal phosphoprotein P0* (*RPLP0)*. Gene sequences and accession numbers are presented in Table 2.

### 2.12. Western Blotting and Immunodetection

Human ASCs (seeded 3160 cells/cm^2^ in 6-well plate) were starved for 24 hours in BM, OM, or AM containing 1% human serum before the 7d inhibitor-supplemented culture, which was also conducted in starvation media. Samples lysed with 2X LAEMMLI sample buffer were analyzed with Western blotting (WB) as described earlier [34]. Briefly, samples were separated with SDS electrophoresis and transferred into polyvinylidene fluoride membrane (0.2 *μ*m PVDF Single application; Bio-Rad, Hercules, CA, USA). Membrane was blocked with 5% milk in Tris-buffered saline supplemented with 0.05% Tween 20 (Sigma-Aldrich). Membranes were incubated with primary antibodies followed by secondary antibody incubation and chemiluminescence detection (ECL Prime Western Blotting Detection Reagent; GE Healthcare, Little Chalfont, UK) and visualized with Chemi Doc MP System (Bio-Rad). Antibodies and dilutions are presented in Table 3.

### 2.13. Statistical Analysis

All results are represented as mean and standard deviation (SD). Statistical analyses were conducted using GraphPad Prism 5 (La Jolla, CA, USA). Statistical differences between the inhibitor-treated samples and the respective controls were tested using the nonparametric Mann–Whitney test followed by Bonferroni post hoc test. Statistical differences with *p* < 0.05 were considered significant. Detailed information of the biological and technical replicates used in statistical analysis is given in Table S2.

## 3. Results

### 3.1. Characterization of hASCs

Surface marker expression of hASCs was analyzed by flow cytometry. The hASCs were characterized as MSCs due to positive expression of CD73, CD90, and CD105; lack of CD3, CD11, CD14, CD19, CD45, CD80, CD86, and HLA-DR expression; and moderate expression of CD34 and CD54 (Table S3).

### 3.2. Inhibition of FAK, ROCK, and ERK1/2 Activity Reduces Proliferation of hASCs

**Figure 1:**
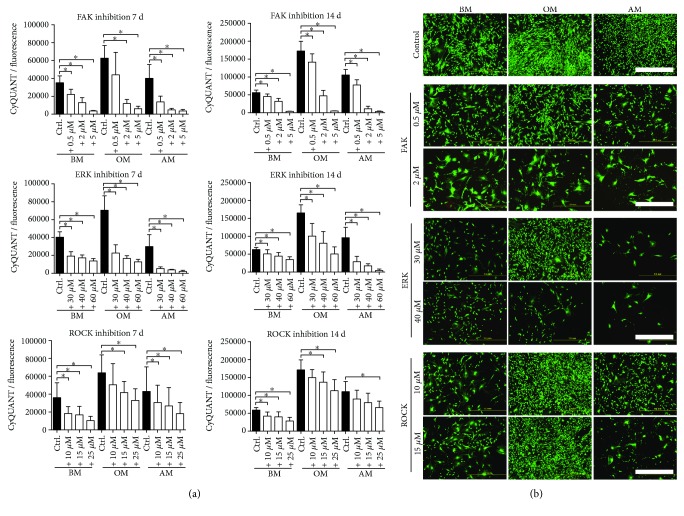
Cell viability and proliferation after 7 and 14 days of culture in response to inhibition of FAK, ERK1/2, and ROCK signaling. (a) hASCs were cultured 7 or 14 d in BM, OM, and AM supplemented with FAK, ERK1/2, and ROCK inhibitors. The inhibitor effect on proliferation was studied in each culture condition separately by comparing the different inhibitor concentrations with the untreated medium control. Significance level 5%, designated with an asterisk (^∗^). FAK, ERK, and ROCK inhibitors: *N* = 12 (independent biological replicates from 4 donors). (b) Representative fluorescence images of LIVE/DEAD-stained hASCs. hASCs were cultured in BM, OM, or AM supplemented with the abovementioned inhibitors. Cell viability was analyzed with LIVE/DEAD assay at 7 d. Green dye represents living cells, red dye dead cells. Scale bar 1.0 mm. BM: basic medium; OM: osteogenic medium; AM: adipogenic medium.

**Figure 2:**
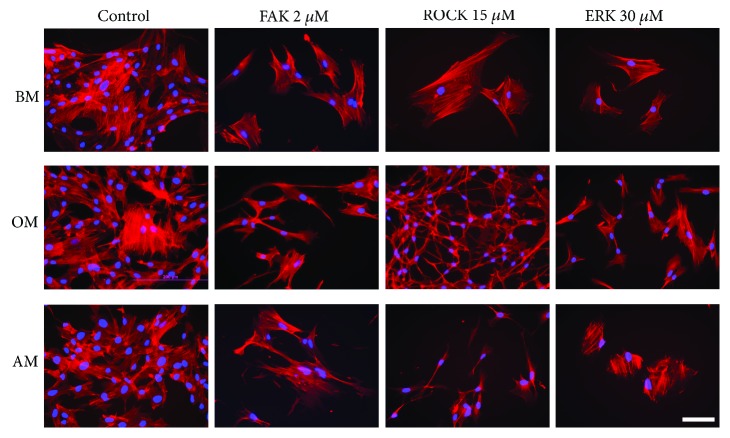
Immunofluorescence staining of actin cytoskeleton and nuclei. hASCs were treated with 2 *μ*M FAK, 15 *μ*M ROCK, or 30 *μ*M ERK inhibitors, and the cytoskeleton was stained with phalloidin (red) and nuclei with DAPI (blue) at day 7. Scale bar 100 *μ*m. BM: basic medium; OM: osteogenic medium; AM: adipogenic medium.

Cell proliferation capacity was evaluated in BM, OM, and AM with gradient concentrations of FAK, ROCK, and ERK inhibitors PF-562271, Y-27632, and PD98059, respectively. CyQUANT assay indicated that the inhibitors have a regulatory function on cell proliferation (Figure 1(a)). The cell numbers were reduced dose dependently in the inhibitor-treated conditions compared to the control conditions. Despite a decrease in the cell number as a response to increased inhibitor concentrations, adherent cells remained viable with a negligible amount of dead cells (Figure 1(b)), as assessed with the LIVE/DEAD method. In addition to the inhibitor function on cell number, the inhibitor treatment also affected the typical fibroblast-like morphology of hASCs. Based on the immunofluorescence staining of actin cytoskeleton (Figure 2), ROCK inhibition caused the most prominent changes to the morphology of the hASCs. Y-27632 treated cells appeared spindle-like in OM and AM media, and the cells in OM had formed a network of star-shaped cells with long extensions.

### 3.3. FAK, ROCK, and ERK1/2 Functions Are Essential to hASC Osteogenesis

**Figure 3:**
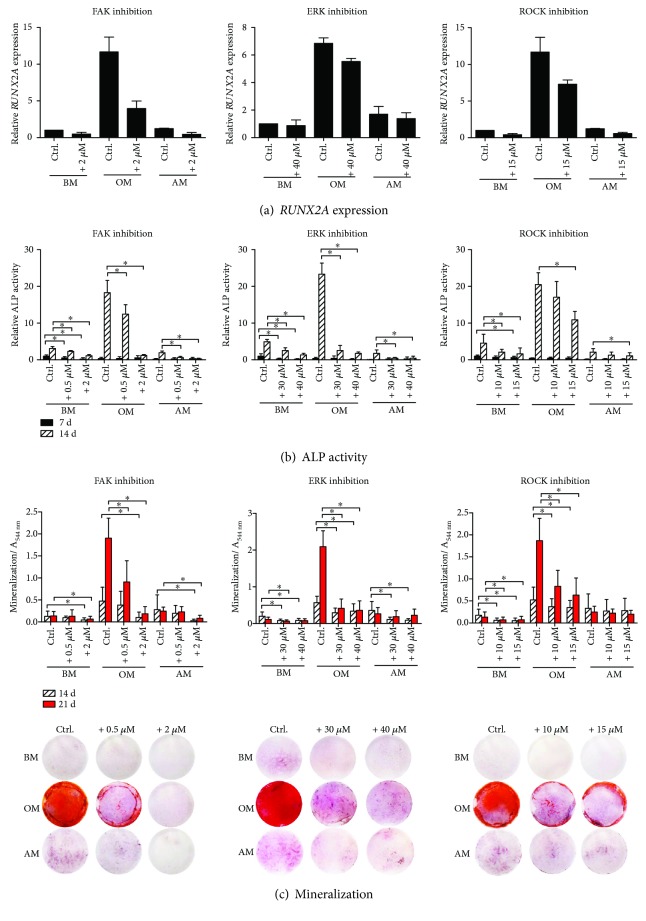
Osteogenic differentiation of hASCs in BM, OM, and AM culture conditions supplemented with FAK, ERK, and ROCK inhibitors. (a) The cells were cultured in BM, OM, or AM supplemented with 2 *μ*M FAK, 40 *μ*M ERK, or 15 *μ*M ROCK inhibitors in addition to medium controls. *RUNX2A* expression was analyzed with qRT-PCR at 7 d. FAK and ROCK: *N* = 5 (independent experiments, 5 donors), ERK: *N* = 3 (independent experiments, 3 donors). (b) ALP activity was analyzed with ALP assay at 7 d and 14 d. The ALP absorbance values were normalized with corresponding CyQUANT results, and the results are presented relative to the 7 d BM sample. Significance level 5%, designated with an asterisk (^∗^). FAK, ERK, ROCK: *N* = 9 (independent biological replicates from 3 donors). (c) Matrix mineralization was analyzed with AR staining after 14 d and 21 d of culture. Quantitative results of AR staining are presented as graphs and corresponding representative images of the stained wells (21 d, area 1.9 cm^2^) are presented below; bright red dye represents mineral. Significance level 5%. FAK, ROCK: *N* = 18 (independent biological replicates from 6 donors, control condition values of the graphs are the same since the experiments were conducted at the same time), ERK: *N* = 15 (independent biological replicates from 5 donors). BM: basic medium; OM: osteogenic medium; AM: adipogenic medium; ALP: alkaline phosphatase; AR: Alizarin Red.

The early osteogenic differentiation potential of hASCs cultured in BM, OM, and AM in the presence or absence of the FAK, ROCK, and ERK inhibitors was assessed by quantitative real-time reverse transcriptase polymerase chain reaction analysis of the bone associated marker gene *RUNX2A* (Figure 3(a)) and by quantitative activity assay of ALP (Figure 3(b)) which is an early marker of osteogenesis [35]. At 7 days of culture, *RUNX2A* expression was markedly upregulated in the OM condition but downregulated by the addition of all studied inhibitors. However, statistical analyses were not done due to the low sample number. The enzymatic activity of ALP was the most prominent in the OM control medium after two weeks of culture, and addition of the inhibitors reduced the enzymatic activity dose dependently. ALP activation was markedly lower in BM and AM conditions, yet a similar trend in the inhibitor effect was seen.

Deposition of calcium phosphate mineral is characteristic to the maturation of osteoblasts and hence, late osteogenic differentiation capacity was studied by Alizarin Red staining protocol after 14 and 21 days of culture. Strong staining for mineralization of the ECM occurred in the OM control medium at 21 d, as indicated by the quantitative analysis and the corresponding red-stained samples (Figure 3(c)). Mineral accumulation was significantly weakened in the inhibitor-supplemented OM conditions. BM and AM, lacking the osteogenic agents, were not able to support matrix mineralization. Although the inhibitors caused statistically significant reduction of mineralization in BM and AM conditions, the absolute values in the control conditions were too low to have any relevance for the mineralization. 2-week culture period was too short for mineral formation in the studied conditions.

### 3.4. Inhibition of FAK and ROCK Enhance Adipogenic Outcome of the hASCs

**Figure 4:**
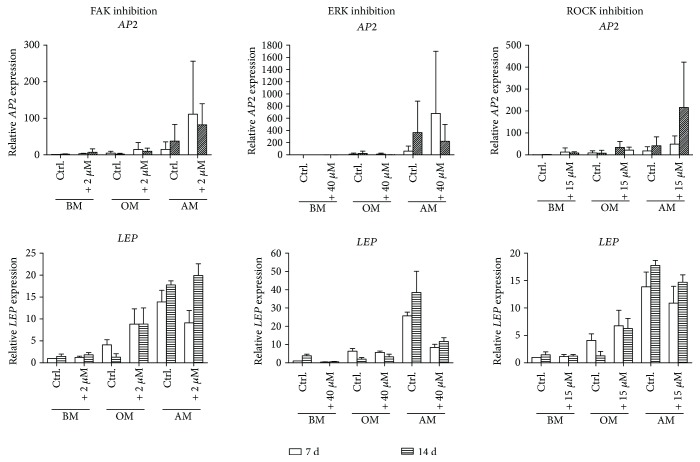
Expression of adipogenic marker genes *AP2* and *LEP* in hASCs treated with FAK, ERK, and ROCK inhibitors. The hASCs were cultured in BM, OM, or AM supplemented with 2 *μ*M FAK, 40 *μ*M ERK, or 15 *μ*M ROCK inhibitors in addition to medium controls. *AP2* and *LEP* expressions were analyzed with qRT-PCR. The expression of *AP2* and *LEP* are normalized with the expression of the housekeeping gene *RPLP0*, and the results are presented relative to the 7 d BM sample. FAK and ROCK: *N* = 5 (independent experiments, 5 donors), ERK: *N* = 3 (independent experiments, 3 donors). BM: basic medium; OM: osteogenic medium; AM: adipogenic medium.

Adipogenic differentiation was analyzed in terms of the expression profiles of adipogenic marker genes *AP2* [36] and *LEP* [37] (Figure 4). As expected, the expression of adipogenic marker genes was most elevated in the hASCs cultured in AM. Based on our results, FAK inhibition increased the expression of *AP2* at both time points in all culture conditions. FAK inhibition also upregulated *LEP* in OM condition at both time points, but in AM, *LEP* expression was only induced at day 14. Inhibition of the Rho-ROCK signaling using Y-27632 led to enhanced *AP2* expression but had an opposite downregulating effect on *LEP* in AM condition. ERK inhibition, on the other hand, augmented *AP2* expression at 7 d but downregulated *AP2* on 14 d. Moreover, ERK inhibition also repressed *LEP* expression at both time points in AM condition suggesting that inhibition of ERK predominantly had a repressing effect on these adipogenic marker genes. Statistical analyses were not done due to the low sample number.

**Figure 5:**
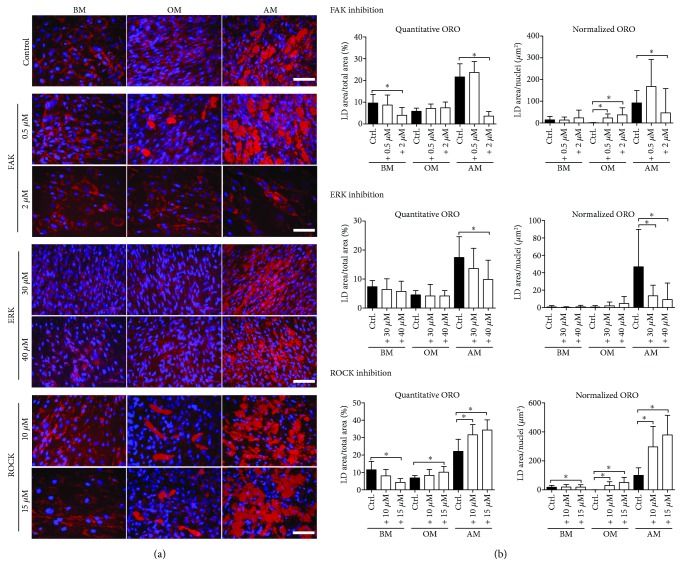
ORO staining of hASCs and quantification of lipid accumulation from ORO-stained fluorescence images. (a) Representative ORO- and DAPI-stained fluorescence images of FAK, ERK, and ROCK inhibitor-treated hASCs at 21 d. Human ASCs were stained with ORO for intracellular lipid accumulation followed by nuclei staining with DAPI. Fluorescence images were taken with Alexa546 for ORO (red) and DAPI (blue) filters. Scale bars 100 *μ*m. (b) ORO-stained samples of FAK, ERK, and ROCK inhibitor-treated hASCs were imaged with fluorescence microscope using Alexa546 and DAPI filters and analyzed with a custom analysis pipeline designed for CellProfiler. Quantitative ORO graph presents the area of all stained LDs as percentages of the total image area. Normalized ORO graph describes LD formation on the single cell level: the area of LD clusters over 10 *μ*m in diameter is normalized with the corresponding nuclei count. Significance level 5%, designated with an asterisk (^∗^). FAK, ROCK: *N* = 13–16 (images from 2 donors), ERK: *N* = 19–21 (images from 3 donors). BM: basic medium; OM: osteogenic medium; AM: adipogenic medium; ORO: Oil Red O; LD: lipid droplet.

To further study adipogenic differentiation of the hASCs, accumulation of lipid droplets was analyzed after three weeks of culture with a fat-soluble diazol dye Oil Red O (ORO), Figure 5(a) [38]. We also quantified the lipid accumulation by creating and optimizing a CellProfiler pipeline to analyze ORO-stained fluorescence images. We analyzed both total lipid droplet area in the cultures (Figure 5(b)) and the area of lipid droplet clusters exceeding 10 *μ*m diameter limit (Figure S1) to visualize the ongoing adipogenic differentiation and maturation of adipocytes which is distinguished by the increasing number of lipid droplets as well as the enlargement of the individual fat vacuoles [39]. Lipid droplet cluster areas over 10 *μ*m in diameter were further normalized with cell nuclei number to obtain results representative of the single-cell level (Figure 5(b)).

Interestingly, FAK inhibitor treatment significantly increased the proportion of large LDs in OM condition. In AM conditions 0.5 *μ*M FAK inhibitor treatment also elevated lipid formation. However, the quantitative results showed that 2 *μ*M FAK inhibitor led to a reduced area of LDs in AM condition. ROCK inhibition resulted in increased adipogenesis on both culture and single-cell level in OM and AM conditions. ERK inhibition reduced the area of ORO-stained LDs in the culture, also when normalized with the cell number.

### 3.5. Western Blot Analysis of the Inhibitor Functionality and Cross Talk between Signal Pathways

**Figure 6:**
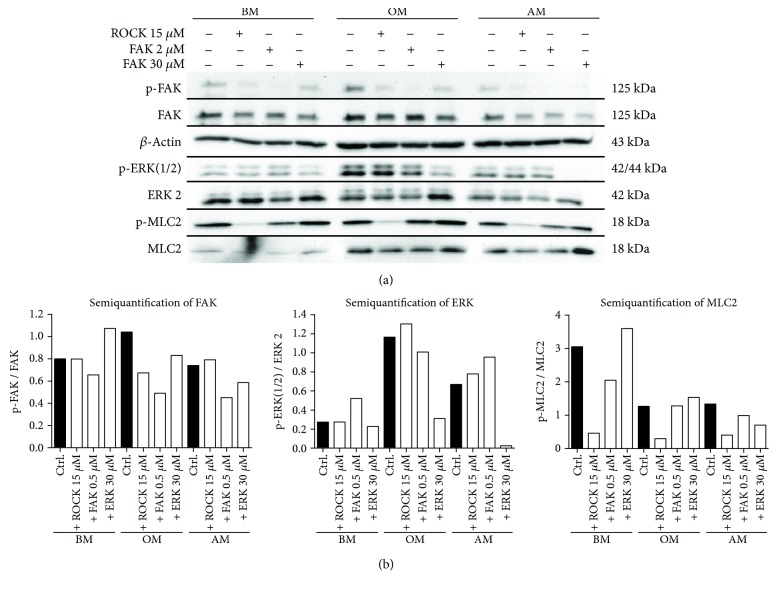
Intracellular protein activation at day 7 as a response to FAK, ROCK, and ERK inhibition. hASCs were cultured 7 days in BM, OM, and AM media containing 1% human serum supplemented with 15 *μ*M ROCK inhibitor, 2 *μ*M FAK inhibitor, or 30 *μ*M ERK inhibitor. (a) Representative WB results of immunoblotted p-FAK, FAK, *β*-actin, p-ERK(1/2), ERK 2, p-MLC2, and MLC2. (b) Semiquantified WB results representing the ratio of phosphorylated and basal form of FAK, ERK, and MLC2 proteins. BM: basic medium; OM: osteogenic medium; AM: adipogenic medium.

The functionality of small molecule inhibitors was confirmed by WB analysis of hASCs cultured in starvation media (Figure 6 and Figure S2). The ratio of phosphorylated and unphosphorylated forms of these proteins was analyzed with semiquantification of the band intensities using ImageJ software [40] (Figure 6(b)). Based on the visual inspection and the semiquantified results, the level of the target protein phosphorylation was clearly decreased by the specific inhibitory molecules confirming the inhibitor functionality.

Furthermore, our results pointed out that FAK, ROCK, and ERK inhibitors affected also other studied phosphoproteins and basal protein levels indicating a prospective cross talk between signaling pathways. For instance, FAK inhibition had a modest decreasing effect on p-ERK1/2 in OM condition and FAK inhibitor also reduced ROCK downstream target p-MLC2 in BM and AM conditions. ROCK inhibition had a complementary decreasing effect on FAK phosphorylation in OM conditions, and also ERK inhibition decreased p-FAK levels in OM and AM conditions.

## 4. Discussion

Despite the fact that hASCs are already used in clinical treatments, the knowledge of the regulatory mechanisms of hASC differentiation originates from research done with varying cell types of human and nonhuman origin. Our aim was to carefully analyze the significance of cell adhesion and cytoskeleton in hASC osteogenic and adipogenic differentiation by using small molecular inhibitors for central proteins in cell adhesion and cytoskeletal dynamics.

Previous studies have noted the importance of FAK signaling in the osteogenic potential of hMSCs [18, 21, 41]. Our results support these findings by showing that the expression of the osteogenic marker gene *RUNX2A*, the enzymatic activity of ALP, and eventually mineralization were distinctly decreased as a result of FAK inhibition in OM condition. In the presence of higher amounts of inhibitor, the cells failed to deposit virtually any mineral, presumably because of the decreased cell number. Role of FAK in the adipogenic differentiation has been investigated mainly with rodent cells and with diverging experimental setups [42–44]. Li and Xie [42] reported that firm adhesion is required for osteogenesis whereas morphological change accompanied with calpain-mediated cleavage of FAK is essential for preadipocytic differentiation and final maturation of adipocytes. On the contrary, a more recent *in vivo* study by Luk and coworkers [44] showed that the adipocyte survival was decreased by FAK knockout. We discovered that in human ASCs, inhibition of FAK activity induced expressions of adipogenic marker genes *AP2* and *LEP* in OM and AM conditions. Moreover, the CellProfiler analysis and normalized lipid droplet values revealed that FAK inhibition significantly induced lipid droplet maturation in OM condition, and 0.5 *μ*M concentration had moderately inducing effect in AM as well. However, the total lipid droplet area in the culture was reduced with FAK inhibition, likely due to the decreased number of adherent cells, since the inhibition also affects the cell adhesion sites. These results together with previous findings suggest that weakening of the adhesion is needed to guide the differentiation towards adipogenic lineage, whereas too robust disruption in the adhesion affects the survival of the cells. Our results support the role of FAK signaling as a central regulator of the differentiation fate of hASCs.

FAK signaling works in cooperation with Rho-ROCK signaling to regulate cytoskeletal dynamics and cell morphology [18, 19]. We found out that both ROCK and FAK inhibition suppresses phospho-MLC2 suggesting that the actin tension is regulated by FAK-ROCK-MLC signaling cascade. Additionally, ROCK inhibition was shown to reduce phospho-FAK levels. Presumably diminished actin tension by ROCK inhibition affects upstream FA assembly and thus levels of FAK protein activation. These findings indicate a bidirectional regulation between actin assembly and cell adhesion mechanisms in hASCs. In addition to the cooperation of FAK and ROCK signaling, Rho-ROCK pathway itself has been shown to be an important regulator of the balance between osteogenesis and adipogenesis in MSCs [7, 13–15]. In the present study, we found out that the functionality of the ROCK signaling cascade was required in the commitment of hASCs to the osteoblastic lineage since inhibition of ROCK signaling caused a dose-dependent decrease of *RUNX2A* expression, reduced ALP activity, and hindered the mineral formation. Our results showed that ROCK inhibition efficiently suppressed the phosphorylation of MLC2, which is involved in actin filament bundling [13]. Thus, hindered actin filament bundling and the disruption of the actomyosin contractility might have led to the observed inhibition of the osteogenesis in our study. Osteogenic differentiation of hMSCs has been demonstrated even without soluble differentiation factors with patterning of the culture platform [7, 45, 46] denoting the importance of the cell shape in the differentiation process. We discovered that the morphology of hASCs was clearly affected by the ROCK inhibitor. Y-27632-treated hASCs appeared spindle-like stellar cells in OM and AM conditions when observed during the culture. These cytoskeletal modifications corresponded to the hindered osteogenic course and likely turned the regulatory switch towards adipogenesis. Indeed, the analysis of the adipogenic gene expression and lipid formation revealed that inhibition of ROCK reciprocally induced substantial adipogenic differentiation in AM but also in the OM condition. These results strongly demonstrate that actin cytoskeleton is an important regulator driving the switch between the osteogenic and adipogenic course of hASC differentiation.

ERK has been suggested to be a mechanosensing protein downstream FAK-Rho-ROCK signaling axis guiding the differentiation fate of hMSCs [23, 24]. In this study, we saw that the inhibition of FAK and ROCK phosphorylation affected phospho-ERK levels suggesting that ERK is regulated in cooperation by the cell adhesion mechanisms and the contractility of the actin cytoskeleton. Interestingly, ERK inhibition also slightly reduced phospho-FAK levels in OM and AM conditions with a currently unknown mechanism. ERK1/2 activity has been linked to the expression of osteogenic markers in hASCs [25] and previous studies have noted that ERK inhibition obstructs osteogenesis in hMSCs [25, 28, 47]. The present study also showed that inhibition of ERK1/2 activation efficiently and dose dependently inhibited the ALP activity and mineral deposition and downregulated *RUNX2A* expression in OM condition. Additionally, ERK1/2 has been suggested to have a regulatory role on MSC adipogenesis, though it has remained contradictory whether the role is activatory or inhibitory [25–28]. ERK1/2 pathway has been suggested to work as a molecular switch between adipogenic and osteogenic lineage commitment of BMSCs when cultured with osteogenic supplements [28]. On the other hand, a more recent study of Xu and coworkers [23] suggested that ERK1/2 is a positive regulator of the hBMSC adipogenesis. Our results consistently indicated that ERK1/2 activity is required for the full osteogenic but also adipogenic potential of hASCs. Inhibition of ERK activity reduced the expression of adipogenic marker genes and lipid accumulation. To our knowledge, this is the first study where ERK inhibition is shown to diminish hASC adipogenesis in both osteogenic and adipogenic culture conditions. Although the proteins investigated in this study are interconnected, the role of ERK in the hASC differentiation was not parallel with the switch-like regulation of FAK and ROCK pathways. However, the mechanism of ERK signaling in adipogenesis needs to be further studied.

## 5. Conclusions

This study set out to determine the significance of cell adhesion and cytoskeletal modifications regulated by FAK and ROCK signaling and their downstream target EKR1/2 for adipogenic and osteogenic differentiation potential of hASCs. The results show that ERK1/2 pathway plays a crucial positive role in both osteogenic and adipogenic courses of hASC differentiation, whereas FAK and ROCK work as molecular switches since they function as positive regulators of osteogenesis but negative regulators of adipogenesis. The investigation of these signaling proteins at the molecular level also highlights the interesting interconnection of FAK, ROCK, and ERK1/2 signaling in hASCs and implicates the complex interplay between these crucial regulators of differentiation fate. This study confirms the molecular mechanisms of cell adhesion and actin tension in human ASCs and gives us tools to modify and guide the cell proliferation and differentiation in stem cell-based applications and therapies.

## Figures and Tables

**Table 1:** Culture media compositions.

Component	BM	OM	AM	Manufacturer
Dulbecco's Modified Eagle Medium/Ham's Nutrient Mixture F-12 (DMEM/F-12)				Thermo Fisher Scientific, Waltham, MA, USA
GlutaMAX	1%	1%	1%	
Insulin	—	—	100 nM	
Human serum (HS)	5%	5%	5%	PAA Laboratories GmbH, Pasching, Austria
Penicillin/streptomycin	1%	1%	1%	Lonza, Basel, Switzerland
L-Ascorbic acid 2-phosphate	—	200 *μ*M	—	Sigma-Aldrich, Saint Louis, MO, USA
*β*-Glycerophosphate	—	10 mM	—	
Dexamethasone (DEX)	—	5 nM	1 *μ*M	
Pantothenate	—	—	17 *μ*M	
Biotin	—	—	33 *μ*M	
3-Isobutyl-1-methylxanthine (IBMX)	—	—	0.25 M	

**Table 2:** The primer sequences and accession numbers for qRT-PCR.

Gene	5′-Sequence-3′	Product size (bp)	Accession number
*AP2*	Forward GGTGGTGGAATGCGTCATG	71	NM_001442
	Reverse CAACGTCCCTTGGCTTATGC		

*LEP*	Forward ACAATTGTCACCAGGATCAATGAC	73	NM_000230
	Reverse TCCAAACCGGTGACTTTCTGT		

*RPLP0*	Forward AATCTCCAGGGGCACCATT	70	NM_001002
	Reverse CGCTGGCTCCCACTTTGT		

*RUNX2A*	Forward CTTCATTCGCCTCACAAACAAC	62	NM_001024630.3
	Reverse TCCTCCTGGAGAAAGTTTGCA		

bp: base pair.

**Table 3:** Primary and secondary antibodies used in Western blot analysis.

Antibody type	Antibody	Host species	Dilution	Incubation
Primary	Anti-*β*-actin^1^	Mouse	1 : 2000	RT, 2 h
Primary	Anti-FAK^2^	Rabbit	1 : 1000	+4°C, overnight
Primary	Anti-p-FAK^2^	Rabbit	1 : 1000	+4°C, overnight
Primary	Anti-ERK2^1^	Rabbit	1 : 1000	RT, 2 h
Primary	Anti-p-ERK1/2^2^	Rabbit	1 : 2000	+4°C, overnight
Primary	Anti-MLC2	Rabbit	1 : 800	+4°C, overnight
Primary	Anti-p-MLC2	Rabbit	1 : 800	+4°C, overnight
Secondary	Anti-rabbit IgG^2^	Goat	1 : 2000	RT, 1 h
Secondary	Anti-mouse IgG^1^	Goat	1 : 2000	RT, 1 h

^1^Santa Cruz Biotechnology, Dallas, Texas, USA. ^2^Cell Signaling Technology, Danvers, Massachusetts, USA.

## Data Availability

The data used to support the findings of this study are available from the corresponding author upon request.

## Supplementary Materials

Supplementary Materials Figure S1: quantification of lipid droplets over 10 *μ*m in diameter from the ORO-stained fluorescence images. Related to Figure 5(b). Figure S2: intracellular protein activation at day 7 as a response to FAK, ROCK, and ERK inhibition. Related to Figure 6. Table S1: donor information. Information of donor gender, age, and adipose tissue harvest site. Related to Materials and Methods section. Table S2: statistical analysis. Information of donor number, biological replicates, and technical replicates of statistical analyses. Related to Section 2.13. Table S3: surface marker expression. Related to Section 3.1.
