# Supplementary material for: Focal Adhesion Kinase and ROCK Signaling Are Switch-Like Regulators of Human Adipose Stem Cell Differentiation towards Osteogenic and Adipogenic Lineages
Source: Stem Cells Int. 2018 Sep 12;2018:2190657. doi: 10.1155/2018/2190657 (PMC6157106; doi:10.1155/2018/2190657)
Supplement: Supplementary Materials — Figure S1: quantification of lipid droplets over 10 μm in diameter from the ORO-stained fluorescence images. Related to Figure 5(b). Figure S2: intracellular protein activation at day 7 as a response to FAK, ROCK, and ERK inhibition. Related to Figure 6. Table S1: donor information. Information of donor gender, age, and adipose tissue harvest site. Related to Materials and Methods section. Table S2: statistical analysis. Information of donor number, biological replicates, and technical replicates of statistical analyses. Related to Section 2.13. Table S3: surface marker expression. Related to Section 3.1. [file 2190657.f1.pdf]

SUPPLEMENTAL MATERIALS AND METHODS

Modules of image quantification pipeline

ColorToGray (input: TIF RGB images, output: OilRedO_orig, DAPI_orig)

The channels of the RGB input images were split into separate grayscale images (pixel size of the images was 0.325 µm). The red channel of the input images was stored as *OilRedO_orig*. The blue channel of the input images was stored as *DAPI_orig*.

MaskImage (input: OilRedO_orig, output: OilRedO)

Some of the images contained a scale bar, which was removed by masking all images with a mask that excludes the scale bar. The masking was applied to *OilRedO_orig* and the resulting images were stored as *OilRedO*.

MaskImage (input: DAPI_orig, output: DAPI)

Some of the images contained a scale bar, which was removed by masking all images with a mask that excludes the scale bar. The masking was applied to *DAPI_orig* and the resulting images were stored as *DAPI*.

Smooth (input: DAPI, output: DAPI_smooth)

Smoothing with a Gaussian filter (typical artifact diameter 15 pixels) was applied to *DAPI* in order to decrease the intensity variation within nuclei for easier segmentation. The resulting images were stored as *DAPI_smooth*.

IdentifyPrimaryObjects (input: DAPI_smooth, output: Nuclei)

Nuclei were segmented from the smoothed DAPI channel using global thresholding based on Otsu's method [33]. Three-class thresholding based on minimization of weighted variance was used and the middle class was assigned to foreground (*i.e.* the nuclei). No additional smoothing was performed. A lower bound of 0.25 was selected for the threshold based on experimentation to avoid false positives in images containing very few or no nuclei. The upper bound was kept at 1.0. Clumped nuclei were detected based on the locations of local intensity maxima and they were separated by applying the Watershed method on the intensity image [34]. No additional smoothing was performed and a minimum distance of 15 pixels between the local maxima was used in this step. The local maxima were detected using a lower-resolution image for faster computation. Holes in the identified objects were filled after both thresholding and declumping. Detected nuclei with a diameter under 15 pixels or over 100 pixels were discarded. Nuclei touching the borders of the image were not discarded. The final nuclei objects were stored as *Nuclei*.

CorrectIlluminationCalculate (input: OilRedO, output: OilRedO_illum)

Background fluorescence and illumination variation in the Oil Red O channel was removed using a polynomial background fit computed separately for each image. The estimated background was stored as *OilRedO_illum*.

CorrectIlluminationApply (input: OilRedO, illumination function: OilRedO_illum, output: OilRedO_corrected)

The estimated background was subtracted from each image and the background-corrected images was stored as *OilRedO_corrected*.

ApplyThreshold (input: OilRedO_corrected, output: OilRedO_binary)

Segmentation of lipid areas from the background-corrected Oil Red O images in *OilRedO_corrected* was performed using global two-class thresholding based on minimization of weighted variance via Otsu's method [33]. No additional smoothing was used. A lower bound of 0.08 was selected for the threshold based on experimentation to avoid false positive lipid areas in images that contain weak or no Oil Red O staining. The upper bound was kept at 1.0. The resulting binary segmentation was stored as *OilRedO_binary*.

Morph (input: OilRedO_binary, output: OilRedO_binary_opening)

Exclusion of lipid areas in *OilRedO_binary* smaller than a given threshold was performed using a binary morphological opening. The opening was performed once with a disk-shaped structuring element, whose diameter was 30.8 pixels (that is 10 µm). The resulting binary images, which only contained lipid areas larger than the given diameter, were stored as *OilRedO-_binary_opening*.

MeasureImageAreaOccupied (input: OilRedO_binary, OilRedO_binary_opening)

The total number of pixels covered by lipid areas of any size (*OilRedO_binary*) and lipid areas larger than the given threshold (*OilRedO_binary_opening*) was calculated.

ExportToSpreadsheet

Image filenames, the total number of pixels, the number of pixels covered by lipid areas of any size, the number of pixels covered by lipid areas larger than the selected threshold and the number of nuclei in all the analyzed images was exported into a spreadsheet.

SaveImages (input: OilRedO_binary)

The binary images representing the segmented lipid regions were stored in 8-bit integer TIF format to allow visual confirmation of accurate segmentation.

SUPPLEMENTAL FIGURES

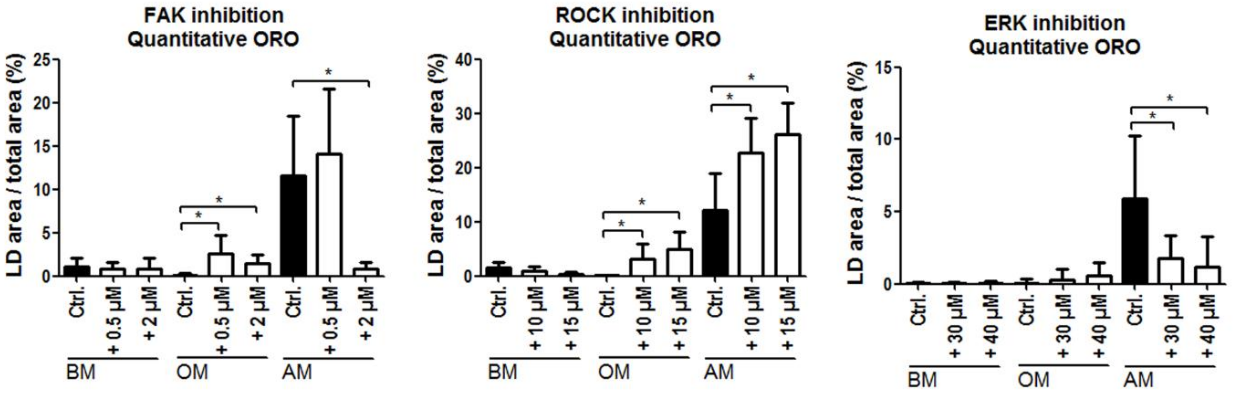

Figure S1. Quantification of lipid droplets over 10 µm in diameter from the ORO-stained fluorescence images. ORO-stained samples of FAK, ROCK and ERK inhibitor-treated hASCs were imaged with fluorescence microscope using Alexa546 and DAPI filters, and analyzed with a custom analysis pipeline designed for CellProfiler (described in main text). Graphs represent the lipid droplet clusters exceeding 10 µm diameter limit as percentages of the total image area. Significance level 5 %. FAK, ROCK: N=13-16 (images from 2 donors), ERK: N=19-21 (images from 3 donors). Abbreviations: LD, lipid droplet; basic medium, BM; osteogenic medium, OM; adipogenic medium, AM.

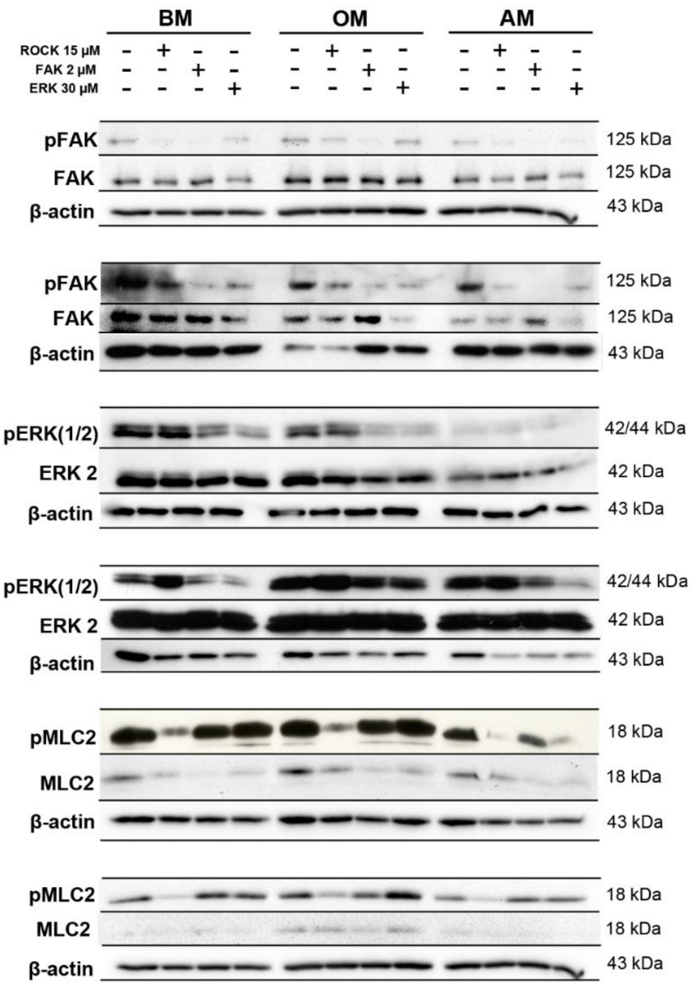

Figure S2. Intracellular protein activation at day 7 as a response to FAK, ROCK and ERK inhibition. Results of two individual experiments (hASC lines) per each pair of phosphorylated and basal proteins. β-actin is used as a loading control. hASCs were cultured 7 days in BM, OM and AM media containing 1 % human serum supplemented with 15 µM ROCK inhibitor, 2 µM FAK inhibitor or 30 µM ERK inhibitor. Samples were analyzed by Western Blotting and immunodetection of p-FAK, FAK, β-actin, p-ERK(1/2), ERK 2, p-MLC2 and MLC2. Abbreviations: basic medium, BM; osteogenic medium, OM; adipogenic medium, AM.

SUPPLEMENTAL TABLES

Table S1. Donor information.

Cell line	Donor gender	Donor age	Harvesting site
**HFSC 1/13**	Female	55	subcutaneous fat from abdomen
**HFSC 8/13**	Female	43	subcutaneous fat from breast
**HFSC 9/13**	Female	55	subcutaneous fat from abdomen
**HFSC 10/13**	Female	51	subcutaneous fat from abdomen
**HFSC 11/13**	Female	40	subcutaneous fat from thigh/femur
**HFSC 1/14**	Female	33	subcutaneous fat from abdomen

Abbreviation: HFSC, human fat stem cell.

Table S2. Statistical analysis. Information of donor number, biological replicates and technical replicates of statistical analyses.

Analysis	Donors (cell lines)	Biological replicates (wells)	Technical replicates
**CyQUANT**	4	12	36
**ALP**	3	9	27
**Alizarin Red**	FAK, ROCK: 6	18	54
	ERK: 5	15	45
**ORO image analysis**	FAK, ROCK: 2	4	13-16 (images)
	ERK: 3	6	19-21 (images)

Table S3. Surface marker expression.

Antigen	Surface protein	Surface marker expression	Fluorophore	Manufacturer
**CD3**	T-cell co-receptor	0.3 ± 0.1	phycoerythrin (PE)	BD Biosciences, Franklin Lakes, NJ, USA
**CD11a**	Integrin alpha L (Lymphocyte function-associated antigen 1)	0.5 ± 0.2	allophycocyanin (APC)	R&D Systems Inc., Minneapolis, MN, USA
**CD14**	Lipopolysaccharide receptor	0.9 ± 0.4	phycoerythrin-cyanine (PECy7)	BD Biosciences
**CD19**	B lymphocyte-lineage differentiation antigen	0.7 ± 0.3	PECy7	BD Biosciences
**CD34**	Hematopoietic progenitor cell antigen 1	12.6 ± 9.7	APC	Immunotools GmbH, Friesoythe, Germany
**CD45**	RO isoform of leucocyte common antigen	1.7 ± 0.9	APC	BD Biosciences
**CD54**	Intercellular adhesion molecule 1 (ICAM-1)	10.4 ± 12.0	fluorescein isothiocyanate (FITC)	BD Biosciences
**CD73**	Ecto-5'-nucleotidase	91.6 ± 8.6	PE	BD Biosciences
**CD80**	B lymphocyte activation antigen (B7-1)	0.6 ± 0.2	PE	R&D Systems Inc.
**CD86**	B lymphocyte activation antigen (B7-2)	0.8 ± 0.5	PE	R&D Systems Inc.
**CD90**	Thy-1 (T cell surface glycoproteins)	96.4 ± 4.9	APC	BD Biosciences
**CD105**	SH-2, Endoglin	98.4 ± 1.0	PE	R&D Systems Inc.
**HLA-DR**	Major histocompatibility class II antigen (MHC-II)	0.9 ± 0.6	PE	Immunotools GmbH
